# Follow-up study of vonoprazan maintenance therapy for reflux esophagitis: A 96-week evaluation in patients with PPI-refractory disease

**DOI:** 10.3892/br.2025.2038

**Published:** 2025-07-30

**Authors:** Hideki Mizuno, Shingo Inagaki, Kazutoshi Yamada, Shinji Kamiyamamoto

**Affiliations:** Department of Gastroenterology, Toyama City Hospital, Toyama 939-8511, Japan

**Keywords:** vonoprazan, gastroesophageal reflux disease, maintenance treatment, recurrent reflux esophagitis

## Abstract

Vonoprazan is a potassium-competitive acid blocker that provides more potent and sustained acid suppression than conventional proton pump inhibitors (PPIs). Vonoprazan may serve a role in the long-term management of gastroesophageal reflux disease (GERD), particularly in patients with reflux esophagitis (RE) who are unresponsive to PPIs. The present study aimed to evaluate the safety, tolerability and efficacy of vonoprazan over a 96-week period in patients with PPI-refractory RE. Initially, 74 patients received vonoprazan 20 mg once daily for 4 weeks. Patients who demonstrated mucosal healing transitioned to vonoprazan 10 mg once daily for a 48-week maintenance phase. Of these, 43 patients continued therapy for an additional 48 weeks. Endoscopic evaluation, symptom scores using the Frequency Scale for the Symptoms of GERD and serum gastrin levels were monitored to assess treatment outcomes and safety. By the end of the 96-week maintenance period, 85.7% of patients who completed follow-up showed no recurrence of mucosal lesions. Among those who discontinued therapy following symptom resolution, 45.8% experienced symptom relapse; however, these patients responded well to reintroduction of vonoprazan. Although serum gastrin levels in the continuous maintenance therapy remained elevated, no adverse events such as carcinoid tumors were reported. These findings suggested that vonoprazan was both effective and well-tolerated as a long-term maintenance therapy for RE and may serve as a viable on-demand treatment strategy for relapse management. While the results are promising, they stem from a highly selected population. Therefore, further randomized, controlled trials are warranted to confirm the generalizability and long-term safety of vonoprazan in broader GERD populations.

## Introduction

Gastroesophageal reflux disease (GERD) is a notable global health concern. As of 2024, the global prevalence of GERD is ~13.9%, affecting nearly 1.03 billion individuals worldwide. However, prevalence rates exhibit notable regional variation: Rates range from 8.7 to 33.1% in the Middle East, 8.8 to 25.9% in Europe and 2.5 to 7.8% in East Asia (1). This increasing trend in GERD prevalence is attributed to multiple factors, including shifts in dietary and lifestyle habits, declining rates of *Helicobacter pylori* infection, enhanced diagnostic capability and evolving criteria in the endoscopic evaluation of reflux-related pathology (2,3). Reflux esophagitis (RE), the erosive form of GERD, is now estimated to affect ~10% of the adult population (4,5). GERD is characterized by the abnormal reflux of gastric content into the esophagus, resulting in symptoms (primarily heartburn and regurgitation) that can disrupt daily functioning and impair quality of life (QOL). It may also present with mucosal injury or both symptomatology and endoscopic abnormality (2,6). Clinically, GERD is broadly categorized into two forms: RE, which is defined by visible mucosal damage on endoscopy, and non-erosive reflux disease, where patients report similar symptoms despite the absence of endoscopic findings (7). The primary goals of GERD management are sustained symptom relief, improved QOL and preventing complications such as anemia, esophageal strictures, Barrett's esophagus and esophageal adenocarcinoma (8). Guidelines for gastroesophageal reflux disease 2021 recommend an individualized treatment approach based on disease severity (2). Proton pump inhibitors (PPIs) or potassium-competitive acid blocker (P-CABs) are typically used as initial therapy, with P-CABs often preferred in more severe or refractory cases. In patients with severe RE, long-term maintenance therapy is generally recommended to prevent recurrence and complications. By contrast, individuals with mild RE may benefit from an on-demand or intermittent treatment strategy. Previous studies (9,10) have demonstrated that progression of mild RE [particularly Los Angeles (LA) classification grades A and B] is relatively infrequent. Mucosal deterioration is observed in ~10% of untreated cases over time, suggesting a generally stable course in the absence of ongoing mucosal injury.

Vonoprazan, a P-CAB developed in Japan, represents a class of acid-suppressing agents that reversibly inhibit the H^+^/K^+^-ATPase enzyme in gastric parietal cells by competitively binding the potassium site, thereby blocking the final step of acid secretion (11-15). By contrast with PPIs, vonoprazan does not require activation in an acidic environment and is unaffected by Cytochrome P450 2C19 (CYP2C19) polymorphisms (12). These pharmacological characteristics contribute to its more rapid, consistent and sustained acid suppression. Its high acid dissociation constant (9.06) facilitates selective accumulation in the acidic canaliculi of parietal cells, supporting a prolonged duration of action and effective acid control from the first dose (11-15). Clinical studies have demonstrated that vonoprazan provides more potent and sustained acid suppression compared with conventional PPIs (13-17). Multiple clinical trials have confirmed the efficacy of vonoprazan (20 mg) in patients with PPI-refractory RE, demonstrating faster symptom relief and superior mucosal healing compared with lansoprazole (13,18). At 8 weeks, vonoprazan (20 mg) has shown healing rates that are 3.0-8.5% higher than those of lansoprazole (30 mg), and up to 1.1-fold greater efficacy in cases of severe erosive esophagitis. In PPI-refractory populations, vonoprazan is associated with a 2-3-fold improvement in mucosal healing compared with historical data on PPIs (19,20). Studies have further supported the effectiveness of vonoprazan (20 mg) in most patients with PPI-refractory RE (21-23). In addition to mucosal healing, vonoprazan has demonstrated more rapid and sustained relief of persistent heartburn symptoms compared with lansoprazole (18-20). Our previous study assessed the efficacy of vonoprazan (10 mg) administered once daily as maintenance therapy over a 48-week period in patients with PPI-refractory RE who achieved mucosal healing following vonoprazan (20 mg) induction therapy (24). The maintenance regimen demonstrated high efficacy, with an endoscopic non-recurrence rate of 88.0% at week 48, and symptom control was maintained in the majority of patients. These results highlighted the potential of vonoprazan (10 mg) as a viable long-term maintenance strategy for individuals unresponsive to standard PPI treatment (24). Despite its proven efficacy in healing erosive esophagitis and its superiority in PPI-refractory cases, data on vonoprazan long-term use in patients who experience symptom or mucosal relapse following initial healing remain limited (25). A previous study (26) have addressed outcomes in patients who relapse following treatment discontinuation and require retreatment or dose adjustment.

Therefore, the present study aimed to evaluate the long-term effectiveness of vonoprazan in maintaining remission and preventing recurrence in patients with erosive esophagitis, particularly those who had previously achieved mucosal healing but later relapsed. This represents a critical unmet need in GERD management and may provide evidence to guide therapeutic strategies for difficult-to-treat cases.

## Materials and methods

### Study design

This was an open-label, single-center follow-up study extending from our previous prospective investigation (24), conducted to evaluate the efficacy of vonoprazan over a 96-week maintenance period in patients with healed RE. The study was performed at Toyama City Hospital (Toyama, Japan) between March 2016 and March 2019. The present follow-up analysis included only patients who had successfully completed the initial 48-week vonoprazan maintenance therapy without discontinuation due to adverse events, mucosal relapse or loss to follow-up. The study protocol was approved by the Ethics Committee of Toyama City Hospital (approval no. 2014-21). Prior to enrollment, all participants received a comprehensive explanation of the study purpose, procedures, potential risks and anticipated benefits. Written informed consent was obtained in accordance with the principles outlined in the Declaration of Helsinki.

### Patients and treatment

Inclusion criteria were as follows: Age ≥20 years; endoscopically confirmed RE classified as LA grades A-D (27) prior to PPI therapy; diagnosis of PPI-refractory RE, defined by a total score ≥8 on the Frequency Scale for the Symptoms of GERD (FSSG) (28) despite receiving standard-dose PPI therapy (rabeprazole 10 mg/day, omeprazole 20 mg/day, esomeprazole 20 mg/day or lansoprazole 30 mg/day) for ≥8 weeks and documented endoscopic healing of erosive lesions following a 4-week course of vonoprazan (20 mg/day).

Exclusion criteria were as follows: Notable organ dysfunction; malignancy or coexisting gastrointestinal disorder, including esophageal stricture, achalasia, eosinophilic esophagitis, inflammatory bowel disease, primary esophageal motility disorders, Zollinger-Ellison syndrome or malabsorption syndromes.

**Figure 1:**
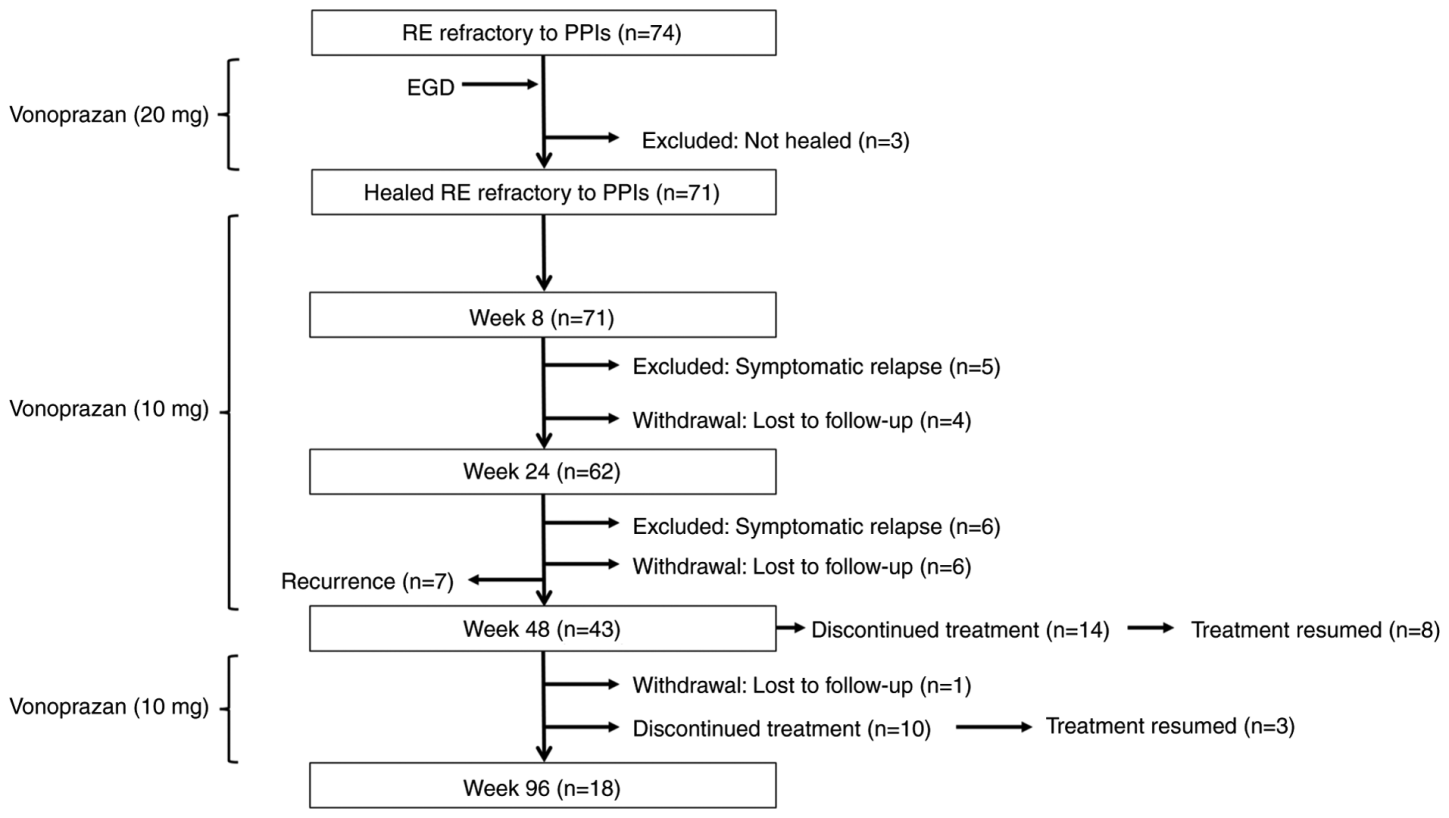
Flow chart of study design. Symptomatic recurrence was defined as acid reflux-related symptoms (3 consecutive days of moderate/severe heartburn and acid regurgitation). RE, reflux esophagitis; PPI, proton pump inhibitor; EGD, esophagogastroduodenoscopy.

Initially, 71 patients (Age, 28-85 years, male/female 34/37) with PPI-refractory RE who achieved mucosal healing following 4-week induction therapy with vonoprazan (20 mg/day) were enrolled in a 48-week maintenance phase with vonoprazan (10 mg/day; Fig. 1). Of these, 50 patients successfully completed the full 48-week regimen, however, seven patients were excluded from the final analysis due to recurrence of erosive lesions: Four with LA grade A, one with grade B and two with grade C. The remaining 43 patients were enrolled in the 96-week maintenance phase. All patients received vonoprazan (10 mg) once daily, with the option to temporarily discontinue medication if symptoms resolved. Patients who elected to pause treatment were monitored on an outpatient basis every 8-12 weeks for up to 96 weeks. In cases of symptomatic relapse or endoscopic evidence of mucosal recurrence, therapy was reinitiated with vonoprazan (20 mg) once daily. Morning fasting serum gastrin levels were measured either at the end of the vonoprazan (10 mg) maintenance phase or at the 96-week time point to monitor hypergastrinemia, a known pharmacological effect associated with prolonged potent acid suppression (29). These measurements also served to assess the safety profile of long-term vonoprazan therapy and explore potential associations between gastrin levels, symptom recurrence and mucosal healing outcomes. *H. pylori* infection was assessed using serum anti-*H. pylori* IgG antibody titers, measured by the *H. pylori*-LATEX ‘SEIKEN’ assay (Denka Seiken Co., Ltd.), according to the manufacturer's instructions. A cut-off value of ≥10 U/ml was used to define *H. pylori* seropositivity.

### Endoscopic evaluation and study endpoints

At the time of diagnosis and during follow-up, upper gastrointestinal endoscopy was performed by four experienced endoscopists to assess mucosal recurrence following maintenance therapy. The grading of erosive esophagitis was independently confirmed by two endoscopists to ensure diagnostic consistency. The severity of esophagitis was evaluated using a modified LA classification system (27). LA classification system is based on the length and extent of mucosal breaks observed during upper gastrointestinal endoscopy and offers a reproducible framework for both clinical diagnosis and therapeutic evaluation. The present study employed a modified LA classification that included the standard LA grades A to D, as well as two additional non-erosive categories: Grade M, indicating minimal changes such as erythema or whitish turbidity and grade N, indicating no visible mucosal abnormalities (30,31). This expanded classification allowed more comprehensive assessment of the full spectrum of mucosal findings. Healing of reflux esophagitis was defined as an endoscopic assessment of grade N or M, reflecting the absence of erosive lesions.

Hiatal hernia was diagnosed when retroflexed endoscopic examination under gastric insufflation revealed a widened esophageal hiatus with visualization of squamous epithelium below the diaphragmatic impression (32).

The presence of atrophic gastritis was assessed endoscopically using the Kimura-Takemoto classification system, which categorizes the extent of gastric mucosal atrophy into six stages: C-0 for no atrophy; C-1 and C-2 for mild atrophy; C-3 and O-1 for moderate atrophy; and O-2 and O-3 for severe atrophy (33). The staging of atrophic gastritis was independently confirmed by two endoscopists to ensure diagnostic consistency.

Upper gastrointestinal endoscopy was performed at the end of the treatment or at 96 weeks to evaluate the presence or absence of recurrent esophageal mucosal erosion. The primary endpoint was the endoscopic remission rate, defined as the proportion of patients maintaining mucosal healing (grade N or M) throughout the 96-week follow-up period.

### Symptom evaluation and study endpoints

GERD symptoms were assessed using FSSG (28), a validated questionnaire comprising 12 items, each scored on a 5-point Likert scale: 0=never, 1=occasionally, 2=sometimes, 3=often and 4=always. The items are grouped into two subscales: Acid reflux-related symptoms and dysmotility-related symptoms. A cumulative score ≥8 is considered indicative of GERD or RE (28).

Participants completed the FSSG questionnaire at the end of the treatment period or at the 96-week follow-up. The results were compared with baseline scores obtained prior to the initiation of maintenance therapy. Symptomatic improvement was defined as a total FSSG score equal to or lower than the baseline value. The rates of symptom improvement were analyzed separately for acid reflux-symptoms and dysmotility-related symptoms to assess therapeutic response. Symptom recurrence was specifically evaluated based on acid reflux-related complaints (2) and defined as the presence of moderate to severe heartburn and acid regurgitation persisting for 3 consecutive days during the maintenance phase. In addition, symptom recurrence was corroborated by an increase in the total FSSG score above the clinical threshold of ≥8, consistent with validated GERD symptom assessment tools (28). In cases of symptomatic exacerbation or endoscopic recurrence of mucosal lesions, vonoprazan (20 mg) once daily was reintroduced as re-treatment. The secondary endpoint was to assess the efficacy of vonoprazan in patients who experienced recurrent RE during the follow-up period.

### Statistical analysis

Efficacy parameters were analyzed in the per-protocol (PP) population. Continuous variables are presented as median and ranges, while categorical variables are expressed as frequency and percentages. The Mann-Whitney U test was used to compare continuous variables, and the χ^2^ or Fisher's exact test was used for categorical variables, as appropriate. Kaplan-Meier analysis was used to estimate the time to first symptom relapse, based on FSSG subscale score during the maintenance phase. Patients who were lost to follow-up or discontinued therapy for reasons unrelated to relapse were censored in the analysis. Changes in FSSG scores from baseline to follow-up were analyzed using paired t-test, with a two-sided significance level of 5%. Data are presented as the mean ± SD. All statistical analyses were performed using IBM SPSS Statistics for Windows, version 29.0 (IBM Corp.).

## Results

### Study profile

A total of 43 patients were enrolled in the 96-week maintenance phase (Fig. 1). Of these, 14 patients (32.6%) experienced symptoms resolution and elected to discontinue treatment during the early phase of follow-up. The remaining 29 patients continued vonoprazan (10 mg) once daily as maintenance therapy. During the maintenance phase, an additional 10 patients (23.3%) discontinued treatment following symptom resolution, and one patient (2.3%) was lost to follow-up. A total of 18 patients (41.9%) completed the full 96-week maintenance regimen with vonoprazan.

### Patient demographics and clinical characteristics [intention-to-treat (ITT) population]

A total of 43 patients were included in the ITT population. Of these, 17 were male, representing 39.5% of the cohort (Table I). The mean age of the ITT population was 66.3±11.9 years, and the mean BMI was 23.6±3.7 kg/m². Prior to initiation of vonoprazan (20 mg) therapy, the distribution of LA classification was as follows: Grade A in 72.1% of patients, grade B in 11.6%, grade C in 9.3% and grade D in 7.0%. The mean disease duration was 19.3±10.8 months. *H. pylori* infection, as determined by serological testing, was present in 32.6% of patients. Gastrointestinal comorbidities were observed in 51.2% of patients, with the most common being hiatal hernia (32.6%) and atrophic gastritis (20.9%; Table I). In addition to baseline comorbidities, the mean fasting serum gastrin levels at the end of the maintenance phase were 1,029.8±751.7 pg/ml (Table I). On-demand therapy was used by 26 patients, representing 60.5% of the study population. Overall, the study cohort was composed primarily of older adults (aged ≥65 years) with mild to moderate reflux esophagitis and a relatively high prevalence of gastrointestinal comorbidity, reflecting a representative clinical population for evaluating the long-term efficacy and safety of vonoprazan (Table I).

### Endoscopic outcomes of maintenance therapy (PP population)

**Figure 2:**
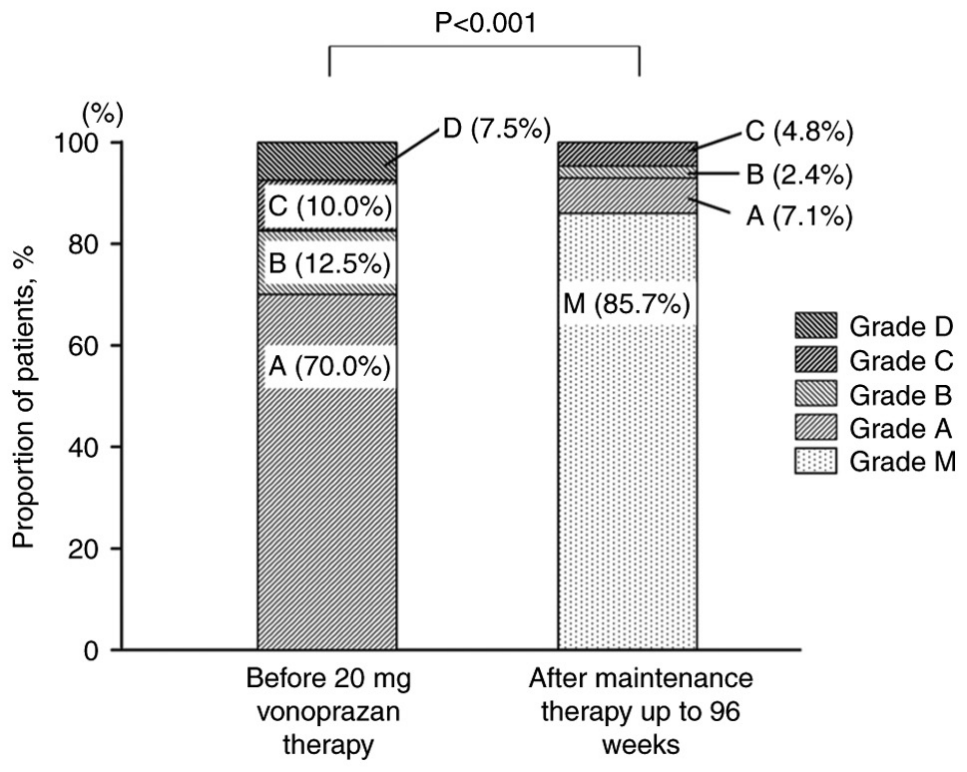
Distribution of Los Angeles classification grades before starting PPI therapy and following maintenance therapy with vonoprazan (10 mg) up to 96 weeks (per-protocol population). PPI, proton pump inhibitor.

At the end of the 96-week maintenance period, 36/42 patients in the PP population were endoscopically classified as grade M, corresponding to a mucosal non-recurrence rate of 85.7% (Fig. 2). Of the six patients who experienced recurrence of esophageal mucosal erosion, the severity was classified as grade A in three cases, B in one case and C in two cases. These findings suggest that long-term maintenance therapy with vonoprazan (10 mg) once daily was effective in sustaining mucosal healing in the majority of patients.

### Efficacy of maintenance therapy: GERD symptoms and gastrointestinal QoL (PP population)

**Figure 3:**
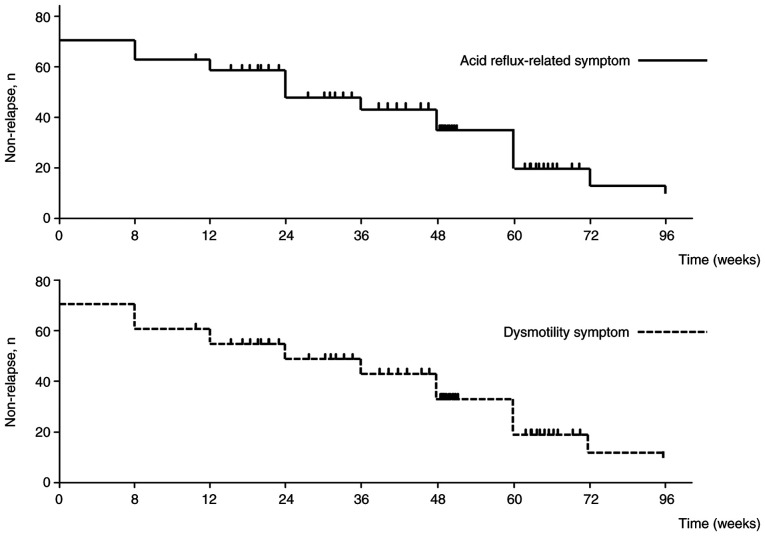
Kaplan-Meier curves depicting patients who did not experience symptom relapse over the 96-week vonoprazan maintenance period. Patients who were lost to follow-up or voluntarily discontinued therapy without documented symptom recurrence were censored at their last observation point.

The symptom non-relapse rates for acid reflux-related symptoms at weeks 8, 24, 48 and 96 were 88.4, 76.7, 69.8 and 55.8%, respectively (Fig. 3). For dysmotility-related symptoms, the corresponding rates were 86.0, 79.1, 65.1 and 51.2%. These data indicated that vonoprazan maintenance therapy offered strong initial symptomatic control, however, its effectiveness in maintaining relief from GERD symptoms gradually declines over time. This trend underscores the importance of individualized, long-term management strategies for optimizing patient outcomes.

### Efficacy of vonoprazan in patients with symptom relapse following completion of treatment (PP population)

**Figure 4:**
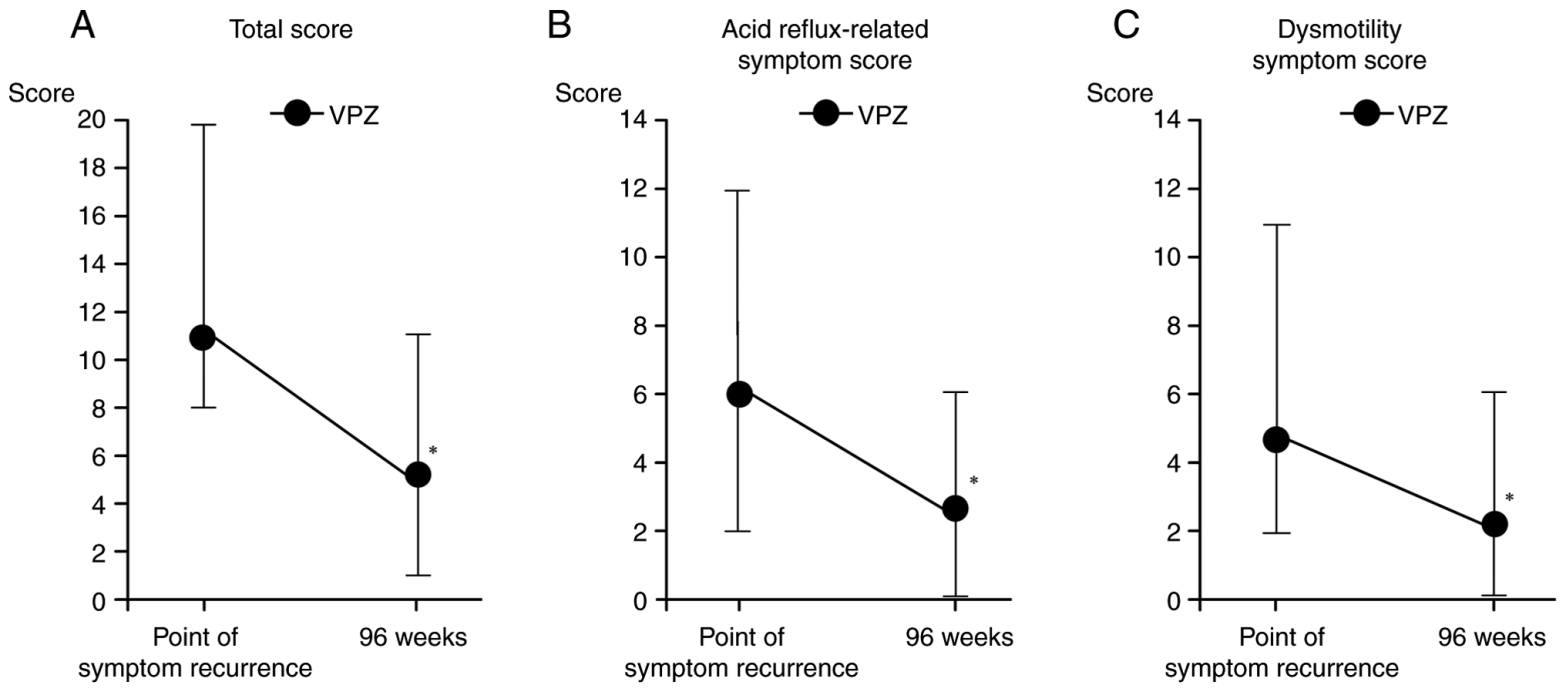
FSSG scores before and after VPZ (20 mg) retreatment in patients with symptom recurrence (n=11). Changes in (A) total, (B) acid reflux-related and (C) dysmotility symptom scores of the FSSG. ^*^P<0.05 vs. point of symptom recurrence. FSSG, Frequency Scale for the Symptoms of gastroesophageal reflux disease; VPZ, vonoprazan.

A total of 18 patients completed the full 96-week course of vonoprazan maintenance therapy. Among the remaining patients, 24 elected to discontinue treatment following symptom resolution, with a mean treatment duration of 56.6±12.4 weeks (range, 48-82 weeks). The mean fasting serum gastrin concentration was 1,130.9±831.0 pg/ml in the continuous maintenance therapy group and 864.7±748.5 pg/ml in the discontinuous maintenance therapy group (Table II). Although serum gastrin levels tended to be higher in patients who experienced relapse, the difference was not statistically significant. These findings suggest that fasting gastrin concentrations were not strongly associated with either clinical or endoscopic recurrence following discontinuation of vonoprazan therapy (Table II). Among the 11 patients who experienced symptom recurrence following the discontinuation of vonoprazan therapy, the mean duration of drug withdrawal prior to relapse was 22.3±9.5 weeks (range, 8-38 weeks; Table III). The mean age of these patients was 65.8±12.2 years, with a mean BMI of 22.8±3.6 kg/m². *H. pylori* infection was present in 27.3% of cases. Gastrointestinal comorbidities were reported in 45.5% of patients, including atrophic gastritis in 9.1% and hiatal hernia in 36.4%. Endoscopic recurrence of esophageal mucosal erosion was observed in 5/11 patients who experienced symptom relapse following discontinuation of vonoprazan therapy. The severity of mucosal relapse based on the LA classification was distributed as follows: Grade A, n=2; B, n=1 and C, n=2. All 11 patients were re-treated with vonoprazan (20 mg) once daily. Following re-initiation of therapy, symptomatic improvement was achieved in all cases and treatment was subsequently discontinued following a mean retreatment duration of 21.5±9.6 weeks (range, 6-40 weeks; Table III). These findings suggest that recurrence may occur even several months after initial drug withdrawal, particularly in patients with anatomical or mucosal risk factors such as hiatal hernia. Nevertheless, the high success rate of retreatment highlighted the efficacy of vonoprazan in managing recurrent GERD symptoms following treatment discontinuation. At the time of symptom recurrence, the mean total FSSG score was 10.8±4.4, with acid reflux-related and dysmotility symptom subscale scores of 6.0±3.1 and 4.8±2.9, respectively. Following retreatment, these scores decreased significantly to 5.1±2.9 (total), 2.6±1.6 (acid reflux-related) and 2.5±1.9 (dysmotility-related; Fig. 4A-C). The decreases in total, acid reflux- and dysmotility-related symptom scores were significant (P=0.002, 0.004 and 0.037, respectively), supporting the robust symptomatic efficacy of vonoprazan following re-administration. These results reinforce the use of vonoprazan not only as an effective long-term maintenance therapy but also as a flexible and reliable rescue treatment strategy for patients who relapse following therapy discontinuation.

### Safety profile

Throughout the 96-week study period, no serious adverse events were observed. Treatment-related adverse effects associated with vonoprazan (10 mg) occurred in 3/43 patients (7.0%), all of whom experienced mild constipation. These symptoms resolved spontaneously during continued administration, and no additional therapeutic intervention was required. Although gastrin levels were elevated [a known pharmacodynamic effect of potent acid suppression (34)], no cases of hypergastrinemia-related complications, including carcinoid tumors, were identified. These findings indicated that while vonoprazan induces sustained elevations in serum gastrin during long-term use, the increase remained clinically benign within the 96-week observation period. This supported the safety of extended vonoprazan maintenance therapy and efficacy in a real-world clinical setting.

## Discussion

To the best of our knowledge, the present study is the first to evaluate the clinical effectiveness of vonoprazan re-treatment in patients who experience symptom recurrence following completion of long-term maintenance therapy, offering novel insights into relapse management strategies in GERD. The present findings demonstrated that on-demand or intermittent use of vonoprazan (10 mg) once daily maintained endoscopic remission in 85.7% of patients over a 96-week period. Furthermore, re-initiation of vonoprazan (20 mg) once daily in patients with symptomatic or endoscopic relapse resulted in rapid and effective resolution of both symptoms and mucosal erosion. These results highlighted the dual therapeutic utility of vonoprazan as a potent and durable long-term maintenance agent and as a reliable rescue therapy following recurrence, even in patients previously exposed to treatment. This flexibility may support individualized treatment approaches, particularly in patients requiring intermittent acid suppression tailored to fluctuating symptom severity or risk of mucosal relapse. The 96-week observation period was selected to capture long-term maintenance outcomes that extend beyond the conventional 6-12-month follow-up commonly reported in GERD studies (2,35). This extended duration was designed to assess the durability of mucosal healing and the incidence of symptomatic or endoscopic recurrence over time. The 96-week framework also allowed for assessment of vonoprazan performance in patients who temporarily discontinued therapy or required re-treatment during the follow-up period.

GERD is a chronic condition with a high tendency to recur, often necessitating long-term maintenance therapy to prevent symptom relapse and mucosal deterioration. The principal therapeutic goals in GERD management include sustained symptom relief and continued healing of esophageal mucosa (36,37). Previous studies have reported that >70% of patients experience symptom recurrence within 6-12 months following discontinuation of initial therapy, underscoring the persistent and relapsing nature of the disease (38,39). The present study further confirmed the therapeutic efficacy of vonoprazan in the management of recurrent reflux esophagitis. The ability of vonoprazan to induce symptom resolution and mucosal healing even after prior treatment discontinuation supports its role as a reliable agent in the long-term management of GERD.

The exploratory VISION study evaluated the long-term safety profile of vonoprazan maintenance therapy over a 5-year period in patients with healed erosive esophagitis, demonstrating its tolerability and sustained efficacy (34). Emerging evidence from suggests that long-term therapy with vonoprazan provides superior control of GERD symptoms and mucosal healing compared with conventional PPIs (34,40). By contrast with large-scale investigations such as the VISION trial, which evaluated vonoprazan in a broad population of patients with healed erosive esophagitis, the present study focused on patients with PPI-refractory disease. It further explored the clinical outcomes of a personalized management approach, incorporating long-term maintenance therapy, on-demand use and retreatment strategies. This tailored framework offers practical insight into real-world therapeutic flexibility and supports the use of vonoprazan in managing patients at higher risk of relapse or requiring individualized dosing regimens.

The safety profiles of vonoprazan at both 10 and 20 mg are comparable with that of lansoprazole (15 mg) in patients with healed erosive esophagitis (41,42). In the present study, on-demand administration of vonoprazan (10 mg) was effective in maintaining endoscopic remission within a 96-week period, with a mucosal non-recurrence rate of 85.0%. These findings support the feasibility of an individualized, symptom-guided dosing strategy for select patients. The cohort analyzed in the present study represented a treatment-responsive and tolerant subgroup, as it includes only those patients who completed the initial 48-week vonoprazan maintenance therapy without significant adverse events or therapeutic failure. As such, the findings primarily reflect the long-term efficacy and safety of vonoprazan in responders, rather than the general population with PPI-refractory reflux esophagitis. Furthermore, Oshima *et al* (18) demonstrated that vonoprazan (20 mg) once daily provided significantly faster and more complete relief of heartburn during the first week of treatment compared with lansoprazole (30 mg) once daily in patients with erosive esophagitis. Together, these data underscore the potent acid suppression, rapid onset of action and safety of vonoprazan, making it a valuable therapeutic option across various GERD treatment scenarios, including both maintenance and rescue settings.

In the present study, 11 patients required re-treated with vonoprazan (20 mg) once daily following symptom recurrence. Symptom resolution was achieved following a median treatment duration of 25 weeks. A subset of patients with GERD experience spontaneous symptom resolution or maintain symptom control without continuous medication (9,43-46). In the present study, 74.4% of patients who achieved symptom resolution maintained good symptom control without ongoing pharmacological therapy. A large cohort study reported that 24-26% of patients with mild erosive esophagitis and 16-18% with severe erosive esophagitis remained off medication during each year of a 4-year follow-up period (47). In the present cohort, 11/24 patients (45.8%) who discontinued vonoprazan experienced symptom recurrence. This recurrence rate is considerably lower than that reported in a systematic review of 19 studies, which found a mean recurrence rate of 75% (95% CI: 68-82%) in patients receiving placebo over follow-up periods ranging from 6 months to 5 years (48). The relatively low recurrence rate in the present study is likely attributable to the robust mucosal healing achieved during the initial induction phase with vonoprazan (20 mg). This observation aligns with findings from Hsu *et al* (49), who reported that longer and more potent acid suppression during the initial treatment phase significantly decreased the risk of recurrence during maintenance therapy. Therefore, the strong initial activity of vonoprazan serves an important role in both the initial and maintenance therapy. During the initial treatment phase, its rapid and potent acid suppression facilitates faster mucosal healing and symptom relief, which is key for patients with PPI-refractory disease. In the maintenance phase, vonoprazan sustains intragastric pH levels >4 for extended durations, thereby minimizing acid exposure to the esophageal mucosa and decreasing the risk of mucosal relapse. This dual-phase pharmacological profile makes vonoprazan particularly well-suited for comprehensive management of GERD, serving effectively as both an aggressive induction agent and a long-term maintenance option. Its consistent acid suppression is especially advantageous in patients with fluctuating symptom patterns or anatomical risk factors such as hiatal hernia, where stable pH control is essential to maintaining clinical remission.

During the course of long-term maintenance therapy, some patients elected to use vonoprazan (20 mg) on an on-demand basis. Among the 18 patients who completed the 96-week maintenance period, eight adopted this strategy and achieved satisfactory symptom control without requiring daily medication. Various therapeutic strategies have been proposed for the long-term management of GERD, including continuous therapy, where PPIs are taken daily, and several non-continuous approaches. These include on-demand therapy, in which PPIs are used only when symptoms arise and discontinued upon relief; intermittent therapy, typically consisting of short treatment courses (1-2 weeks) in response to symptom recurrence, and threshold therapy, where the interval between doses is progressively extended as long as symptoms remain absent (38). The present findings support the feasibility of a flexible, symptom-guided approach using vonoprazan, particularly in patients with stable or mild disease following mucosal healing, and underscore its potential use within personalized GERD treatment paradigms. A key advantage of on-demand therapy is its efficiency in decreasing medication burden: Studies (38,50) have shown that total PPI consumption in this group is approximately half that of patients receiving continuous therapy. In a real-world survey evaluating long-term PPI maintenance therapy for GERD, no significant differences were observed among continuous, on-demand and intermittent therapy groups with regard to patient-reported outcomes, including symptom control, satisfaction or preference for the type of maintenance regimen (39). Patients with a longer duration of GERD were more likely to adopt non-continuous strategies, including on-demand use (39). Moreover, patients in the non-continuous therapy group demonstrated higher awareness of the potential adverse effects associated with long-term PPI use compared with those on continuous therapy (39). On-demand therapy with vonoprazan is an effective alternative maintenance strategy for patients with mild RE (51,52). These findings suggest that on-demand therapy not only decreases medication exposure but may align better with patient preferences and safety awareness, especially in individuals with chronic GERD who seek to minimize long-term pharmacological dependency. The present study implemented an individualized on-demand approach by monitoring patient adherence through the remaining amount of vonoprazan tablets at each follow-up visit. A total of 26/43 patients (60.5%) elected to discontinue regular therapy, indicating successful symptom control without daily medication. This approach is cost-effective, as it minimizes unnecessary prescriptions and decreases overall drug consumption. The findings further support the utility of a flexible, patient-centered dosing strategy (particularly on-demand use), which can achieve sustained symptom control in a substantial proportion of patients. Such strategies not only lessen medication burden but also improve cost-efficiency without compromising therapeutic efficacy, making them a viable option for long-term GERD management in appropriately selected individuals.

Vonoprazan is distinguished by its potent and rapid acid-suppressive properties, which offer notable therapeutic advantages in the treatment of GERD. However, such notable acid inhibition has raised concerns regarding long-term safety, particularly the potential risk of carcinoid tumor development associated with sustained hypergastrinemia (53). Despite these concerns, vonoprazan has consistently demonstrated a favorable safety and tolerability profile in multiple clinical studies (19,21-23,34,40). Notably, a 5-year clinical trial found no histological evidence of hypergastrinemia-associated changes in the gastric mucosa, including neuroendocrine cell hyperplasia or carcinoid tumor formation (34). These findings reinforce the long-term safety of vonoprazan, even during extended use, and support its suitability as a maintenance therapy for GERD. In the present study, the mean morning fasting serum gastrin levels measured following a treatment regimen consisting of vonoprazan (20 mg) for 4 weeks followed by 10 mg for 96 weeks was 1,029.8 pg/ml. This elevation is consistent with the pharmacological profile of vonoprazan, which is known to induce hypergastrinemia due to sustained acid suppression (34). Monitoring of serum gastrin levels is an integral component of the safety evaluation and provided supporting evidence that long-term vonoprazan therapy, including on-demand or retreatment use, did not result in clinically notable hypergastrinemia-associated complications in the present cohort (34,40). This degree of hypergastrinemia is anticipated, given the potent and prolonged acid suppression of vonoprazan, which leads to compensatory gastrin secretion via negative feedback mechanisms (54). Elevated gastrin levels stimulate enterochromaffin-like cell proliferation, raising concerns regarding the potential development of gastric neuroendocrine tumors or mucosal hyperplasia (34). However, clinical evidence suggests that this elevation does not translate into clinically meaningful pathological changes within a 2-year observation period (25,52). In the present cohort, no histological abnormality, carcinoid tumors or serious gastric mucosal events were observed. These results are consistent with findings from the 5-year VISION study, which also reported no hypergastrinemia-related neoplasia despite sustained gastrin elevation (34). Clinically, this implies that while serum gastrin levels should be monitored during long-term vonoprazan therapy (particularly in high-risk individuals) the elevations observed in typical patients with GERD are physiologically adaptive and not inherently harmful. Nonetheless, ongoing surveillance in larger, more diverse populations and extended follow-up durations is key to exclude any potential late-emerging risks.

The present study had limitations. First, the single-center design and small sample size may limit the generalizability of the findings to broader GERD populations. Second, the open-label nature of the study introduced potential patient-associated bias in symptom reporting and treatment adherence. Third, objective physiological assessment of acid suppression, such as 24-h esophageal pH monitoring, was not performed; inclusion of such measures would have strengthened the association between pharmacological effect and clinical outcomes. Fourth, due to the non-randomized and observational design, the potential for selection bias and confounding cannot be excluded. Additionally, a key limitation was the enrichment of the analyzed population with patients who had already demonstrated clinical benefit and tolerability during a preceding 48-week course of vonoprazan maintenance therapy. As a result, the findings primarily reflect outcomes in a responder subgroup and may not be fully generalizable to all patients with PPI-refractory erosive esophagitis. Despite these limitations, the present study provided real-world insight into the management of a high-risk GERD population, including patient-driven decisions regarding treatment continuation, discontinuation and re-initiation. Future studies should aim to include larger, more diverse patient cohorts and incorporate randomized, controlled designs with comparator arms to evaluate the long-term efficacy and safety of vonoprazan across the full clinical spectrum of GERD.

In conclusion, long-term maintenance therapy with vonoprazan, particularly when individualized through on-demand use, is both safe and effective in preventing symptom recurrence and mucosal relapse in patients with erosive esophagitis. The present study highlighted the practical utility of vonoprazan as a flexible and reliable treatment option in real-world GERD management. While the results are encouraging, confirmation through larger, multicenter, randomized controlled trials is warranted to validate these findings and broaden their generalizability.

## Figures and Tables

**Table I:** Demographic and clinical characteristics of patients at baseline (ITT population; n=43).

Characteristic	Value
Mean age, years	66.3±11.9
Sex, male/female	17/26
Mean BMI, kg/m^2^	23.6±3.7
BMI ≥25	14 (32.6)
Duration of illness, months (mean ± SD)	19.3±10.8
PPI used before VPZ 20 mg, RPZ/OPZ/EPZ/LPZ	13/6/19/5
Baseline LA classification (grade A/B/C/D)	31/5/4/3
*Helicobacter pylori* infection (%)	14 (32.6)
Gastrointestinal comorbidities, n (%)	22 (51.2)
Atrophy of gastric mucosa, n (%)	9 (20.9)
Esophageal hiatal hernia, n (%)	14 (32.6)
Mean serum gastrin, pg/ml	1,029.8±751.7
On-demand therapy, n (%)	26 (60.5)

ITT population, intention-to-treat population; BMI, body mass index; PPI, proton pump inhibitor; VPZ, vonoprazan; RPZ, rabeprazole; OPZ, omeprazole; EPZ, esomeprazole; LPZ, lansoprazole.

**Table II:** Clinical characteristics between the patients undergoing continuous and discontinuous maintenance therapy.

Characteristic	Continuous maintenance therapy (n=18)	Discontinuous maintenance therapy (n=24)	P-value
Mean age, years	65.2±13.2	67.2±11.2	0.619
Sex, male/female	5/13	11/13	0.192
Mean BMI, kg/m^2^	23.6±3.7	23.1±3.8	0.683
Mean duration of illness, months	22.6±23.4	20.3±21.0	0.735
Baseline LA classification (grade A/B/C/D)	14/2/0/2	16/3/4/1	0.351
*Helicobacter pylori* infection, positive/negative	2/7	5/7	0.161
Atrophy of gastric mucosa, n (%) (closed type/open type)	3 (25.0) 3/0	6 (21.2) 6/0	0.398
Esophageal hiatal hernia (%)	6 (33.3)	8 (33.3)	0.627
Mean serum gastrin, pg/ml	1,130.9±831.0	864.7±748.5	0.346
On-demand therapy, n (%)	8 (44.4)	17 (70.8)	0.081
Mean duration of treatment, weeks	96	56.6±12.4	0.001

BMI, body mass index.

**Table III:** Clinical characteristics of patients with recurrence of symptoms after discontinuation of vonoprazan.

Age, years	Sex	LA grade at recurrence	Duration until recurrence, weeks	Duration of re-treatment of vonoprazan, weeks	BMI kg/m^2^	*Helicobacter* *pylori* infection	Atrophy of gastric mucosa	Esophageal hiatal hernia	FSSG total score at recurrence
68	Female	LA-C	24	24	17.5	Negative	No	No	9
46	Female	LA-M	24	24	32.4	Negative	No	No	9
71	Male	LA-M	20	24	24.9	Positive	No	Yes	8
76	Male	LA-M	32	16	21.9	Negative	No	No	8
68	Male	LA-B	16	32	19.5	Negative	No	Yes	8
80	Female	LA-C	26	20	24.1	Negative	No	Yes	18
59	Male	LA-M	8	40	22.5	Negative	No	No	8
70	Male	LA-A	38	10	28.9	Negative	No	No	10
84	Male	LA-A	14	25	19.6	Negative	Closed type	No	11
53	Female	LA-M	11	6	18.3	Positive	No	Yes	21
49	Female	LA-M	32	16	21.3	Positive	No	No	9

BMI, body mass index; FSSG, frequency scale for the symptoms of gastroesophageal reflux disease.

## Data Availability

The data generated in the present study may be requested from the corresponding author.
